# Decentralized Management of Home Care Services for Seniors: Protocol for a Participatory Action Research

**DOI:** 10.2196/58271

**Published:** 2025-01-17

**Authors:** Virginie Savaria, Johanne Queenton, Annie Carrier

**Affiliations:** 1 Université de Sherbrooke Sherbrooke, QC Canada; 2 Centre de recherche sur le vieillissement Sherbrooke, QC Canada

**Keywords:** health system, decentralization, management, home care services, seniors, collaboration

## Abstract

**Background:**

The centralization of decision-making power in the public health care system has a negative impact on the practice of professionals and the quality of home care services (HCS) for seniors. To improve HCS, decentralized management could be a particularly promising approach. To be effective, strategies designed to incorporate this management approach require attention to 3 elements: autonomy of local stakeholders, individual and organizational capacities, and accountability for actions and decisions. Not many studies have focused on strategies for integrating decentralized and collaborative management at the local level in HCS.

**Objective:**

The overall aim of this study is to coconstruct HCS management strategies and explore decentralized practices in the day-to-day work of low-level managers and professionals. The specific objectives, in collaboration with local HCS stakeholders, are to (1) identify concrete and achievable strategies for decentralized management, and (2) describe factors (facilitators and obstacles) that could potentially influence their integration.

**Methods:**

This participatory action research involves a cyclical process. Before initiating the cycles, a preliminary stage consists of forming a steering committee composed of managers (n=3), professionals (n=3), seniors (n=3), informal caregivers (n=3), and the research team (n=3). This committee will facilitate multistakeholder consultation to coconstruct local management strategies based on a real-life problem identified by the committee. The steering committee will also guide the research process. The first cycle will consist of establishing an initial plan of decentralized management strategies. During the observation phase, meetings of 4 homogeneous focus groups, including managers, professionals, seniors, and informal caregivers, will be held. During the reflection phase, a thematic analysis will be carried out, and data will be interpreted and validated by the steering committee. Then, in the action phase, results will be presented to managers and professionals so that they can coconstruct a plan of decentralized management strategies to prioritize. The second cycle will explore the factors involved. The observation, reflection, and action phases will be repeated. Ultimately, the results of the 2 cycles will be integrated in a model coconstructed by the steering committee.

**Results:**

Data collection is in progress; the partnership officially began on February 1, 2024, and the plan is to continue data collection through 2025. The steering committee will validate the data to ensure that they are accurate and that the results reflect the reality of local stakeholders.

**Conclusions:**

By identifying decentralized and collaborative management strategies at the local level as well as factors to facilitate their integration in HCS, this approach can be used for other decentralized management projects in different areas of the health care system. This study will give decision makers insight into strategies aimed at improving the management of their institution, which will enhance seniors’ well-being and the quality of their health care services.

**International Registered Report Identifier (IRRID):**

DERR1-10.2196/58271

## Introduction

### Background

Over the past 4 decades, health care systems in most countries have been reshaped by reforms based on new public management (NPM) [1]. NPM aims to improve the effectiveness, efficiency, and quality of services by focusing on results-based management and cost control [2]. Inspired by NPM [3,4], the last 2 health and social service reforms in the Canadian province of Québec made a shift toward the centralization of management practices [5,6]. With the aim of improving health system performance, the 2003 [7] and 2015 reforms [8,9] gradually concentrated decision-making power, responsibilities, and control, and moved them from lower management to the senior management level of the health system [3,6]. The trend toward centralized management seems to be continuing with the new 2024 reform [10-12], which will concentrate decision-making power in the hands of a new agency called *Santé Québec* [13]. Such centralization of decision-making power necessarily relies on management by results, accountability, and partial privatization of services [9,14]. Consequently, departmental priorities focus on effectiveness and efficiency [14]. The changes resulting from centralized management can have an impact on the conditions of practice of local stakeholders [14], including low-level managers, professionals, seniors, and informal caregivers. These changes also influence the quality of home care services (HCS) for seniors [14], a sector where demand is increasing as the population ages [15,16]. Centralization reduces the latitude of managers and professionals [5], which in turn is associated with a reduction in how meaningful they find their work [14,17]. Their ability to offer services that are accessible and adapted to the needs of the population diminishes [18-20]. Professionals and seniors agree that the continuity and quality of HCS must be improved [18].

To address the present challenges faced by HCS, decentralization could be a particularly promising approach [5,21]. Decentralization implies the delegation of decision-making, power, and responsibilities [21]. The decentralization process involves reconfiguring relations among the national, regional, and local levels to work toward collaborative vertical and horizontal management [22]. A decentralization process involves decentralized management strategies, which are management practices that promote the delegation of decision-making, power, and responsibilities from a higher to a lower level [23]. To be effective, local decentralized management involves three elements: (1) delegating authority to lower-level managers and professionals, (2) strengthening their individual and organizational capacities, and (3) sharing accountability for decisions to be made [23]. Decentralization has the potential to improve the performance of the health system [21,24], the efficient allocation of material and human resources, and responsiveness to the needs of seniors [22].

Moving toward decentralized management is particularly relevant when we consider the main effects of the current centralized management on the health system in Québec. First, administrative reforms and a centralized management approach can reduce the autonomy of professionals [4,14], whose decisions must be approved by senior management, which delays the taking of concrete action in the field [5,19]. The centralization of decision-making power and formalized communication mechanisms limit direct relationships between different local stakeholders [19]. Second, the standardization of practices and use of statistics have increased the focus on performance optimization (eg, cost-effectiveness) [19]. On the one hand, the management and organizational techniques put in place to optimize the work of professionals (eg, standardized clinical tools) [14] require them to invest a great deal of time in their daily tasks [15,19], which decreases the time they devote to direct care for seniors [14,19]. On the other hand, this standardization leads to a tightening of HCS allocation criteria, which impacts the accessibility of services [19]. Some seniors are being denied services, although they were eligible before the 2015 reform [19]. Third, there is a wide gap between the expectations of professionals and managers [15]. Professionals are mainly concerned with improving the quality of care and services for seniors [15]. Managers are more focused on the performance of HCS. For example, HCS must contribute to reducing hospitalization times and increasing the number of seniors who received services [15]. Fourth, services are developed in silos, without any overall coordination [25,26]. This leads to the duplication of services, blurred roles for each HCS provider, a lack of coordination, and competition between service providers [27]. As a result, overly centralized management has negative effects on the practice of professionals and the quality of services for seniors.

Now that the Québec government has made decentralization a priority [28-30], documenting decentralized management in its HCS becomes relevant. Some studies have examined the integration of decentralization in a health care system but generally only from the viewpoint of evaluating this in a specific context. However, each health system has its own management context and structure [31]. Thus, in today’s hypercentralized system, bringing decision-making closer to Québec managers, professionals, seniors, and informal caregivers requires finding a way to integrate decentralized management strategies at the local level of HCS. This study aims to answer the following question: To integrate decentralized management in HCS effectively, what potential strategies should be used and what factors should be considered? The overall aim of this study is to coconstruct HCS decentralized management strategies in the day-to-day work of low-level managers and professionals. The specific objectives, in collaboration with local HCS stakeholders, are to (1) identify concrete and achievable strategies for decentralized management, and (2) describe factors (facilitators and obstacles) that could potentially influence their integration.

### Theoretical Model

According to the theoretical model of Ohrling and colleagues [24], decentralization is a dynamic process that evolves over time and in which three elements interact: (1) delegation of authority to local stakeholders, (2) strengthening of individual and organizational capacities, and (3) accountability for actions and decisions. *Authority* refers to the different degrees of decision-making power and autonomy delegated to lower levels of managers and professionals [24]. To achieve a balanced distribution of authority at different levels of management, managers need to identify tasks and decisions that can be delegated. To ensure that the selection of priorities is in line with the needs of seniors, managers must have a certain amount of latitude in their decision-making [32]. Thus, strategies that support effective decentralized management involve low-level managers having sufficient authority to select those priorities, identify tasks and decisions that can be delegated, and allocate resources [24]. *Capacities* involve the possibility of strengthening individual and organizational abilities to assume delegated decision-making power and responsibilities [24]. The individual ability of managers and professionals to take the initiative is based on their personal aptitudes [33]. As for organizational capabilities, certain norms and culture help encourage the initiatives of managers and professionals [24]. Finally, managers and professionals need to be *accountable* for their decisions and their impacts on HCS [24]. This element involves specifying how responsibilities will be integrated and distributed between managers at all levels and professionals [24]. Also, to facilitate the delegation of authority and sharing of responsibility, managers and professionals should coordinate services, ensure quality, and meet institutional standards [24]. To be effective, strategies must include a sufficient degree of delegation, combined with the capacity on the part of local stakeholders and institutions to make choices in line with optimized performance and to be accountable for these choices aligned with local needs and priorities [34].

### Literature Review

This study addresses the question: What potential strategies should be used and what factors should be considered to effectively integrate decentralized management into HCS? Based on the 3 elements of the model of effective decentralization (authority, capacity, and accountability) [24], the literature review presents these strategies and factors.

#### Decentralizing Management: Strategies to Be Explored at the Local Level

The main strategy of *authority* involves establishing clear policies that contain guidelines regarding the changes to be made to the degree of autonomy and decision-making power at each management level (provincial, regional, and local) [35-37]. When local stakeholders are involved in developing them, these policies are closer to local needs and improve the quality of services [38]. However, these policies often have gray areas that make them difficult to integrate at the local level [37], such as poor communication between provincial, regional, and local levels regarding policy applications [35,39]. Furthermore, it is important to consider the financial dimension of HCS with respect to managers’ decision-making power over budget allocation [23]. This strategy involves increasing local control and autonomy over budget management, including the choice of how to allocate funds [40,41]. This control enables choices to be made in line with local needs, which leads to more responsive services [41].

*Capacity* strategies comprise ways to increase managers’ individual capabilities. For example, training programs should focus on managerial leadership skills, health management organization, and organizational changes to come with decentralized management strategies [33,35]. To increase organization capacity and sustain local control and autonomy with respect to budget management requires a system for allocating financial resources [37]. Under such a system, the provincial level retains the power to divide tax revenues equitably between various local institutions [40,42].

*Accountability* calls for a variety of strategies that are easier to integrate at the local level and have more lasting positive effects [40]. The most promising of these strategies is, first, the creation of clinical networks or committees that give local stakeholders a voice with HCS senior management [38,43]. Second, one of the most common strategies is to reorganize responsibilities between the central government and the local institutions to increase the latters’ autonomy [24,36,37,41,44,45]. Third, the reorganization of human resources management responsibilities includes increasing the decision-making power of local organizations in recruiting and training professionals [36,37,46]. A fourth strategy is to have provincial and regional levels use a bottom-up approach to plan health priorities that involve local managers and communities in identifying initiatives and projects for strategic planning of HCS [47,48]. A fifth strategy involves local managers using clear report cards that include objectives, concrete strategies, and performance indicators for decentralization-related changes in the institution [24].

#### Factors Influencing the Integration of Decentralized Management Strategies

Factors facilitating (n=8) the integration of decentralized management strategies are presented first, followed by factors representing obstacles (n=7). Regarding *authority*, 2 facilitators encourage the delegation of decision-making power to lower-level managers and professionals [24]. The willingness of managers at different levels to delegate certain tasks and responsibilities encourages their participation in the integration of decentralized management strategies [32]. Coaching senior managers through monthly meetings can help build a relationship of trust between the provincial and local levels [49]. Senior management’s commitment helps motivate lower-level managers to take on accountability [39]. Two facilitators can help strengthen organizational *capacities,* leading to enhanced integration of decentralized management strategies [24]. First, the institution must have the material, human, and financial resources needed to put the strategies into practice [35,46,48,50]. For example, quality equipment (such as a high-performance IT system) [39], the right infrastructure, a qualified workforce, and budget planning facilitate the integration of changes linked to decentralization [35]. Creating a culture of mutual trust within the institution encourages managers and professionals to take the initiative and make decisions [33]. In terms of individual capacities, 2 facilitators reflect the personal skills of managers at different levels and professionals [33]. The first concerns coping strategies for managers and professionals, which are methods they can put in place to help them reorganize their work routines [51]. These methods enable them to spend more time on their new tasks and responsibilities, for instance, by reducing the number of meetings they need to attend [41,51]. Senior managers can help by limiting the amount of bureaucracy they have to deal with [33,52]. The second facilitator is the leadership skills of managers at different levels. These skills can facilitate the integration of decentralization to define new relationships with provincial and regional levels, plan resource allocation, build a new shared vision, and lead change [43,53]. Two facilitators are associated with *accountability*. Clarity of roles and responsibilities between provincial and local levels regarding resource allocation and accountability for action enables proactive integration of decentralized management strategies [33,47]. Collaboration between provincial and local levels facilitates the integration of a budget that respects local needs, strategic direction, and government priorities [46].

Several factors stand in the way of integrating decentralized management strategies. Regarding *authority*, 2 factors make it more difficult for the provincial level to delegate to lower levels [24]. Difficulties communicating between the provincial level and local institutions regarding the execution of a policy or reform can lead to confusion [37,41,51]. As a result, a gap may be created between policy and its implementation [39]. Good communication means that information about the new policy to be implemented is clear, accurate, and transmitted to the right individuals [39]. Moreover, when the provincial level decides to retain a high degree of control over local institutions, it is more difficult to integrate decentralized management strategies [41,47]. Local managers then lack autonomy [41] and decision-making power [36], which makes it difficult to lead change. As for organizational *capacity*, lack of resources and funds can limit the changes to be integrated [46,49,51,52]. Concerning individual capacity, limited managerial skills [49,54], and lack of preparation [48,51] can affect managers’ ability to make decisions in their new role and demotivate them [48]. In addition, 2 obstacles are associated with *accountability* for coordinating quality services aligned with local needs [24]. A lack of clarity in roles and responsibilities is a source of confusion when new responsibilities linked to decentralized management strategies are added [37,38]. Managers and professionals then find it difficult to assume their new responsibilities, for which they may be accountable. The second obstacle concerns budget allocation based on the central government’s political motivations. This factor can create a gap between government funding and actual budgetary needs at the local level [46].

The studies discussed in the aforementioned literature review are useful as they focus on decentralized management strategies and factors likely to influence their integration. However, some of them were carried out in countries where the political, organizational, and legal structures of the health care system differ from those in Canada and Québec [24,29,55]. Furthermore, some studies did not involve the participation of all local stakeholders in developing decentralized management strategies. As a result, none of them fully captures the complexity of the dynamic and evolving process of decentralization and the factors that can influence it in the Québec context.

## Methods

### Design

**Figure 1:**
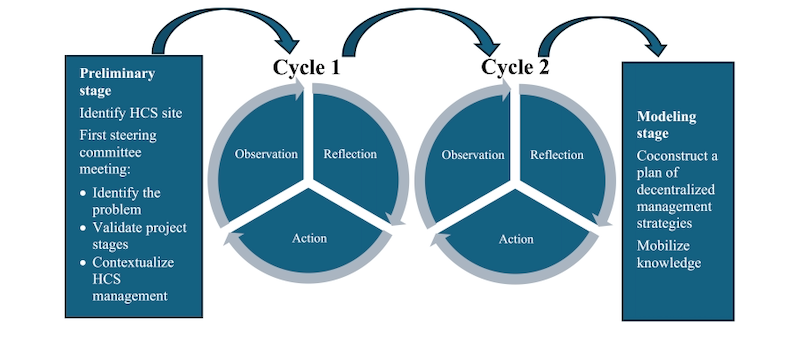
Cyclical process of participatory action research based on the participatory action research model developed by Roy and Prévost [57]. HCS: home care services.

To meet our objectives, a participatory action research (PAR) design was chosen [56]. The PAR design gives HCS stakeholders the power and the tools to explore and coconstruct management strategies adapted to their context and to solve a specific problem that they have identified [56]. The study will start with a preliminary stage, followed by 2 cycles, each composed of 3 phases (observation, reflection, and action), and end with a modeling stage (Figure 1 [57]). The preliminary stage consists of selecting the research site and forming a steering committee. The first cycle will establish a plan of decentralized management strategies (objective 1). The second cycle will consolidate the decentralized management strategy plan and anticipate its integration in HCS by describing factors that could potentially influence the integration of these strategies (objective 2). Finally, the modeling stage will combine the results of the 2 cycles and consolidate the decentralized management strategy plan. A cyclical reflective process is necessary for PAR to ensure constant 2-way feedback between theory and practice [58,59].

### Study Components (Preliminary Stage)

To define the problem and initiate the PAR process, a steering committee will be set up, composed of 3 managers, 3 professionals, 3 seniors, and 3 informal caregivers identified at the site as well as 3 members of the research team (VS, JQ, and AC). Each type of stakeholder will be equally represented to encourage constructive exchanges and ensure that all steering committee participants feel free to express themselves [56]. The committee will enable a concerted stakeholder approach to the study, aimed at coconstructing [56] decentralized management strategies based on a real-life problem identified by the committee. Before starting the PAR cycles, an initial meeting of the steering committee will be held to discuss everyone’s role and involvement [56]. In addition, this meeting will provide an opportunity to contextualize current HCS management and identify a target problem to be addressed in this study. Committee members will be invited to comment on the proposed research process [56]. Members of the research team on the steering committee will play a facilitating role and support the other members in the effective search for solutions [56].

### Cycle 1: Identify Decentralized Management Strategies (Objective 1)

#### Observation Phase: Data Collection

A convenience sample of 24 participants will be recruited to form 4 focus groups of 6 participants each (managers, professionals, seniors, and informal caregivers) to collect data [60]. Each group will be homogeneous to encourage participants to express themselves freely [61]. The qualitative research literature recommends having 6-8 participants in each focus group to ensure interesting exchanges and the opportunity for each participant to answer questions and share their point of view and experience [60]. These focus groups will be used to gather views on decentralized management strategies and the factors influencing their integration as identified in the literature, according to their different status (manager, professional, senior, or informal caregiver) [62]. A member of the steering committee will be present during the discussions to assist the research team. Focus group meetings will last 90 minutes [62]. The aim will be to identify decentralization strategies to solve the target problem. Focus group interviews will be recorded and conducted with the help of a guide [60]. In addition, the student researcher (VS) will document her reflections and the different stages of the study in a research journal [63].

#### Reflection Phase: Data Analysis

First, an initial list of potential codes will be drawn up from the literature review on decentralized management strategies (objective 1), for example, establishing clear policies [64]. Second, initial deductive codes will be generated by identifying the units of meaning linked to a list of codes based on the literature review. The emergent initial codes will be generated by identifying the units of meaning in the data relating to decentralized management strategies that are not included in the list of codes (objective 1). Third, to search for themes, codes will be selected to create potential themes. Relevant coded data extracts will be collected for the created themes [64]. To ensure that the themes and subthemes are meaning-based interpretative stories [65], each will have a definition based on the extracted codes. Fourth, the themes will be examined in relation to the coded data extracts and the data as a whole [64]. Themes and subthemes will be organized in a chart to think creatively and reflexively the data and facilitate communication with steering committee members [56,65]. Fifth, the themes, subthemes, and overall chart will be revised and defined [61]. Sixth, relevant and illustrative data extracts will be selected to represent the themes [64]. The 6 steps of the Braun and Clarke's [65] analysis will be carried out by the student researcher (VS) and validated by 2 members of the research team (AC and JQ). Seventh, a meeting will be held with steering committee members to validate and enrich these analyses. The validation will enable us to make sure that the themes that have been created represent the perspective of field stakeholders [65]. The chart resulting from the data analysis will be presented to the steering committee and will help members reflect on the solutions and decentralized management strategies to be integrated.

#### Action Phase: Identify and Coconstruct Decentralized Management Strategies

During this phase, the results of the reflection phase will be shared with all HCS stakeholders involved in the strategies identified (ie, professionals on the HCS team). These stakeholders will be identified by the steering committee. A meeting will be held with these stakeholders led by the student researcher (VS) and a member of the steering committee. First, the student researcher will present the decentralized management strategies. Then she will invite the stakeholders to prioritize the strategies according to 3 criteria: strategy’s relevance to the problem identified, strategy’s feasibility in terms of accessibility of the necessary resources, and strategy’s acceptability to all stakeholders (managers, professionals, seniors, and informal caregivers) [66]. Finally, a plan of decentralized management strategies will be drawn up with the stakeholders [56,67]. This will identify the decentralized management strategies to be prioritized, the associated tasks, the resources needed, and the time required [56].

### Cycle 2: Identify Factors Influencing the Integration of Decentralized Management Strategies (Objective 2)

#### Observation Phase: Data Collection

With the same 24 participants, meetings of 4 homogeneous focus groups of 6 participants each (managers, professionals, seniors, and informal caregivers), lasting 90 minutes [62], will be conducted. In the second cycle, the focus groups will be used to anticipate and coconstruct factors that could potentially influence the integration of decentralized management strategies in HCS.

#### Reflection Phase: Data Analysis

A thematic analysis using Braun and Clarke’s 6 steps [64] will be conducted to interpret the data collected and will be carried out in the same way as in the first cycle. Both deductive and inductive approaches will be used to define an initial list of potential factor codes based on the literature and to allow new themes to emerge [64]. The analysis will generate a chart of factors linked to the decentralized management strategies identified in the first cycle. A steering committee meeting will then be held to present the chart and validate the factors involved in integrating decentralized management strategies [56].

#### Action Phase: Describe the Factors Influencing Integration of the Strategies

In the second cycle, the action phase will consist of a meeting with the same stakeholders as in the first cycle. This meeting will provide an opportunity to share the factors (facilitators and obstacles) identified and to reflect on those most likely to influence the integration of the decentralized management strategies identified in the first cycle. This discussion will lead stakeholders to consolidate the plan of decentralized management strategies and anticipate their integration [56,67]. Thus, at the end of the 2 cycles, the data collected should enable empirical saturation to be reached, with further collection no longer providing sufficiently new or different information to justify another PAR cycle [68].

#### Modeling Stage

The PAR process will end with a fourth meeting of the steering committee. At this meeting, the results (decentralized management strategy plan and factor figures) will be integrated into a model coconstructed by the steering committee [56,67]. Based on this model, the decentralized management strategy plan will be converted into recommendations regarding which strategies to prioritize to resolve the management issues identified.

### Recruitment and Sampling

The participants will be main HCS stakeholders: managers, professionals, seniors, and informal caregivers. They will be recruited with the help of steering committee members, who will explain the research project to them and verify their interest in participating. Convenience sampling will then be used to target a total of 6 managers, 6 professionals, 6 seniors, and 6 informal caregivers (n=24) [64]. Participants will be recruited according to the following inclusion criteria: (1) they must have received services or worked in HCS for a continuous period of at least 6 months; (2) they must have an interest in HCS management; (3) they must be able to express themselves in French; and (4) they must be able to consent to participate in the study. They will participate in the observation phase in both cycles.

### Ethical Considerations

This study was approved by the research ethics board of the integrated university health and social services center (#2024-5221/Carrier). All participants will be required to provide free and informed written consent for each cycle. Participants will be able to withdraw from the study at any time without any repercussions. They will also be informed of the confidential nature of the data collected and the procedures followed to ensure confidentiality and anonymity. The data collected will be secured (on a password-protected computer or in a locked fireproof filing cabinet at the Research Centre on Aging) and only the research team will have access to them. Members of the steering committee will fill out a collaboration protocol, based on the one developed by Fortier et al [69]. As a PAR, the impact of change on participants must be considered. The study, which is conducted in partnership with the participants [61], who are treated with respect throughout the research process, cannot be used to evaluate their work [61]. Seniors and informal caregivers will receive financial compensation of CAD $25 (US $17.67) for their transportation to a point of service offering HCS.

## Results

The study started in February 2024 and will be carried out according to an optimal schedule over a 1½ period (Table 1). In the preliminary stage, we obtained ethical approval and developed the interview guides. We also developed a research partnership with an HCS site. Initially, one meeting of the steering committee was planned. However, 2 additional meetings were necessary to contextualize the management and organization of the HCS site. The first cycle began in July, with the first focus group in July 2024. We had recruited 12 managers and 7 home care workers. We are currently at the stage of conducting focus groups with seniors and caregivers. Each cycle will last approximately 4 months. To ensure knowledge mobilization, the research team will prepare scientific papers and make presentations. Members of the steering committee will be invited to participate. This study received financial support (June 2023) from a major Canadian funding body, the Social Sciences and Humanities Research Council of Canada (#430-2023-00783/Carrier).

**Table 1:** Tasks and timeline for each cycle of the participatory action research.

Task				Time
**Preliminary stage**				
	Ethics approval		December 2023^a^	
	Development of interview guides		December 2023^a^	
	Development of research partnership		February 2024^a^	
	First meeting of steering committee		March 2024^a^	
	Second and third meetings to contextualized HCS^b^		June-July 2024^a^	
**First cycle**				
	Focus group	July-October 2024^a^		
	Thematic analyses	November-December 2024^a^		
	Fourth (initially second) meeting of steering committee	December 2024^a^		
	Meeting with local stakeholders	January 2025		
**Second cycle**				
	Focus group		January 2025	
	Thematic analyses		February-March 2025	
	Fifth (initially third) meeting of steering committee		March 2024	
	Meeting with local stakeholders		April 2025	
**Modeling stage**				
	Sixth (initially fourth) meeting of steering committee	April 2025		
	Dissemination and mobilization strategies	June-October 2025		

**^a^**Tasks completed at the time of publication of this paper.

^b^HCS: home care services.

## Discussion

### Strengths and Limitations

This study is designed to explore decentralized management strategies that are concrete and adapted to the realities of local stakeholders as well as to anticipate factors involved in integrating the strategies. The methodological choices are scientifically rigorous and will be validated in each phase of the study by members of the steering committee [68,70]. To encourage greater reflexivity on the part of the student researcher (VS), the focus group interviews will be cocoded with at least 1 member of the research team, and the analyses will be validated by the steering committee. The research team’s experience in the health care system is an asset as they are familiar with the management context [61,68,70]. However, decentralized management strategies will be developed in a centralized management context, which could present an obstacle to the integration of some strategies. In addition, given the power relationship between managers and professionals, social desirability is a potential bias. Nonetheless, the views of various local stakeholders will enrich the steering committee’s discussions [56]. In addition, the research team members will play a facilitating role and support other members in effectively identifying decentralized management strategies and the factors involved [56]. The research team will pay attention to power issues during discussions to ensure that all members can express themselves freely [56].

### Broad Implications

The research project will address a gap in the literature by identifying concrete decentralized management strategies adapted to the local reality of an HCS site and anticipating factors involved in integrating these strategies. The results will advance practical knowledge regarding decentralized management, which can serve as a basis for further studies designed to advance knowledge in this field.

This study will provide recommendations for innovative solutions to a management issue identified by an HCS site. It considers the main stakeholders in HCS in the search for innovative solutions and helps identify decentralized management strategies that meet the needs of the institution. The strategies identified can also be adapted to other environments with similar characteristics [71]. Knowledge of decentralized management will be transferred between participants and members of the steering committee [58]. In addition, this project will increase the self-determination of steering committee members through the PAR process, which will encourage them to get involved in the search for strategies [58]. By involving seniors and informal caregivers in the steering committee and focus groups, this study encourages community participation in the search for management strategies. As a result, the decentralized management strategies identified will undoubtedly meet seniors’ needs [72].

## Abbreviations

HCS: home care services
NPM: new public management
PAR: participatory action research
